# Spectral‐Integrated Thermal Absorption Model for Broadband Laser‐Protective Reflectors Under Supercontinuum Irradiation

**DOI:** 10.1002/advs.202518750

**Published:** 2026-02-15

**Authors:** Yukang Feng, Yanzhi Wang, Yulin Zhang, Yesheng Lu, Yu Chen, Fanxin Meng, Huisong Hu, Zhongyang Xing, Jianda Shao

**Affiliations:** ^1^ Laboratory of Thin Film Optics Shanghai Institute of Optics and Fine Mechanics Chinese Academy of Science Shanghai China; ^2^ Center of Materials Science and Optoelectronics Engineering University of Chinese Academy of Sciences Beijing China; ^3^ College of Advanced Interdisciplinary Studies Nanhu Laser Laboratory Hunan Provincial Key Laboratory of High Energy Laser Technology National University of Defense Technology Changsha Hunan China

**Keywords:** laser coatings, supercontinuum laser, temperature rise, thin film design

## Abstract

With the advancement of high‐power multiwavelength lasers, their intense power density causes severe thermal damage to high‐value targets. Nanometer‐scale multilayer coatings with high reflectivity offer a promising means of protection. However, research on their thermal responses under multiwavelength irradiation and broadband protection remains limited. To address this issue, a novel universal laser‐energy absorption model based on spectral intensity integration is proposed for nanometer multilayer coatings. Based on this model, we designed ultrabroadband all‐dielectric nanometer multilayer reflectors with a theoretical average reflectance greater than 99.9% in the 450–1200 nm range, and we experimentally evaluated their temperature under supercontinuum laser irradiation. The proposed energy‐absorption model reveals that the energy absorption in reflectors under laser irradiation follows an exponential decay behavior, which is distinctly characterized by two absorption coefficients. The contribution of short‐wavelength absorption to temperature rise exceeds that of long‐wavelength absorption. Therefore, a short‐wavelength‐prioritized reflector exhibits the smallest temperature increase and can be effectively leveraged for broadband laser protection. The new model allows efficient temperature‐rise simulations of reflectors under laser irradiation using the finite‐element method, with simulation results showing good agreement with the experimental data. These findings provide valuable insights for thermal evaluation of laser‐protective reflectors.

## Graphical abstract

A universal laser absorption model based on spectral integration enables the design of ultrabroadband dielectric multilayer reflectors for laser protection. The model reveals exponential decay of absorption characterized by two coefficients, and a design that prioritizes short‐wavelength reflection minimizes heating. Simulations using the model show good agreement with supercontinuum laser experiments, confirming its effectiveness for broadband laser protection.

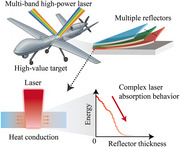

## 1 Introduction

High‐power continuous lasers have emerged as a key focus in modern research owing to their high‐power density [1, 2], energy efficiency, long‐range precision, and near‐instantaneous strike capability [3]. With the rapid advancement toward higher‐power, multiband [4], and multitype systems, the threat posed to high‐value targets, particularly through mechanisms such as thermal damage and material ablation, has become increasingly severe. [5, 6] Therefore, developing effective protection technologies is crucial. Supercontinuum (SC) lasers [7, 8], with an ultrabroad spectral range and high brightness, are widely used in spectroscopy [9, 10, 11] and attoscience [12, 13, 14]. Compared with single‐wavelength continuous waves, these sources pose potentially greater risks to targets because their broadband spectrum interacts with materials whose absorption varies strongly with wavelength.

Various laser‐protection strategies have been explored to date [15, 16, 17, 18, 19, 20, 21]. Nanometer dielectric reflective mirrors, composed of numerous thin layers, are widely employed owing to their low absorption and high damage thresholds [22, 23, 24, 25]. However, conventional nanostructures are often designed with narrow spectral bands and normal incidence features [26]. In practice, laser beams from directed‐energy weapons can operate across multiple wavelengths and strike at varying incident angles [27], placing stringent demands on the spectral and angular robustness of protective coatings. Developing broadband, large‐incidence‐angle, thermally robust protective coatings, as well as constructing accurate thermal response models are critical for enhancing laser defense capabilities. Moreover, despite the high reflectivity of dielectric coatings, a small fraction of incident light, especially under high average power, can still be absorbed by the coating [28]. This localized absorption leads to an increase in temperature, which may cause thermal expansion, surface deformation, and even thermal damage [29, 30]. Although the thermal effects of laser irradiation have been studied both theoretically and experimentally [6, 31, 32, 33, 34, 35, 36], most studies focused on single‐wavelength heating [21, 31, 37, 38, 39] and approximate multilayer coatings either as an effective surface heat source or by treating absorption using the Beer–Lambert law. Such simplifications neglect the interference‐induced standing‐wave field and the layer‐by‐layer refractive‐index contrast in dielectric multilayers. Therefore, they cannot reproduce the wavelength‐dependent, depth‐resolved absorption profile inside the stack. Moreover, existing thermal models rarely incorporate the full spectral characteristics of broadband or multiwavelength lasers, leaving the combined influence of different spectral components and different layer positions largely unexplored. These limitations prevent conventional models from resolving how spectral components interact with depth‐dependent absorption to govern temperature rise, highlighting the need for a general, layer‐resolved absorption model applicable to both single‐wavelength and broadband laser conditions.

Therefore, accurate modeling and quantification of the temperature rise of broadband reflectors under multiband laser irradiation are essential for designing advanced high‐power laser‐protective reflectors. In this study, a novel spectral‐intensity‐integrated, depth‐resolved theoretical absorption model is proposed to analytically compute the wavelength‐dependent local absorption in each layer and integrate it over the entire spectrum. Based on the proposed model, we fabricated ultrabroadband reflectors and evaluated their temperature‐rise trend using an SC‐laser irradiation setup. We calculated the temperature rise of the mirror using the proposed energy‐absorption model and finite‐element analysis. The developed laser‐energy absorption model and temperature‐rise calculation methods can be used to evaluate the thermal behavior of arbitrary multilayer films, thereby expanding their potential application in optical communication, photonic devices, and precision metrology.

## 2 Results and Discussion

### 2.1 Concept of Broadband Protection Reflector

**FIGURE 1:**
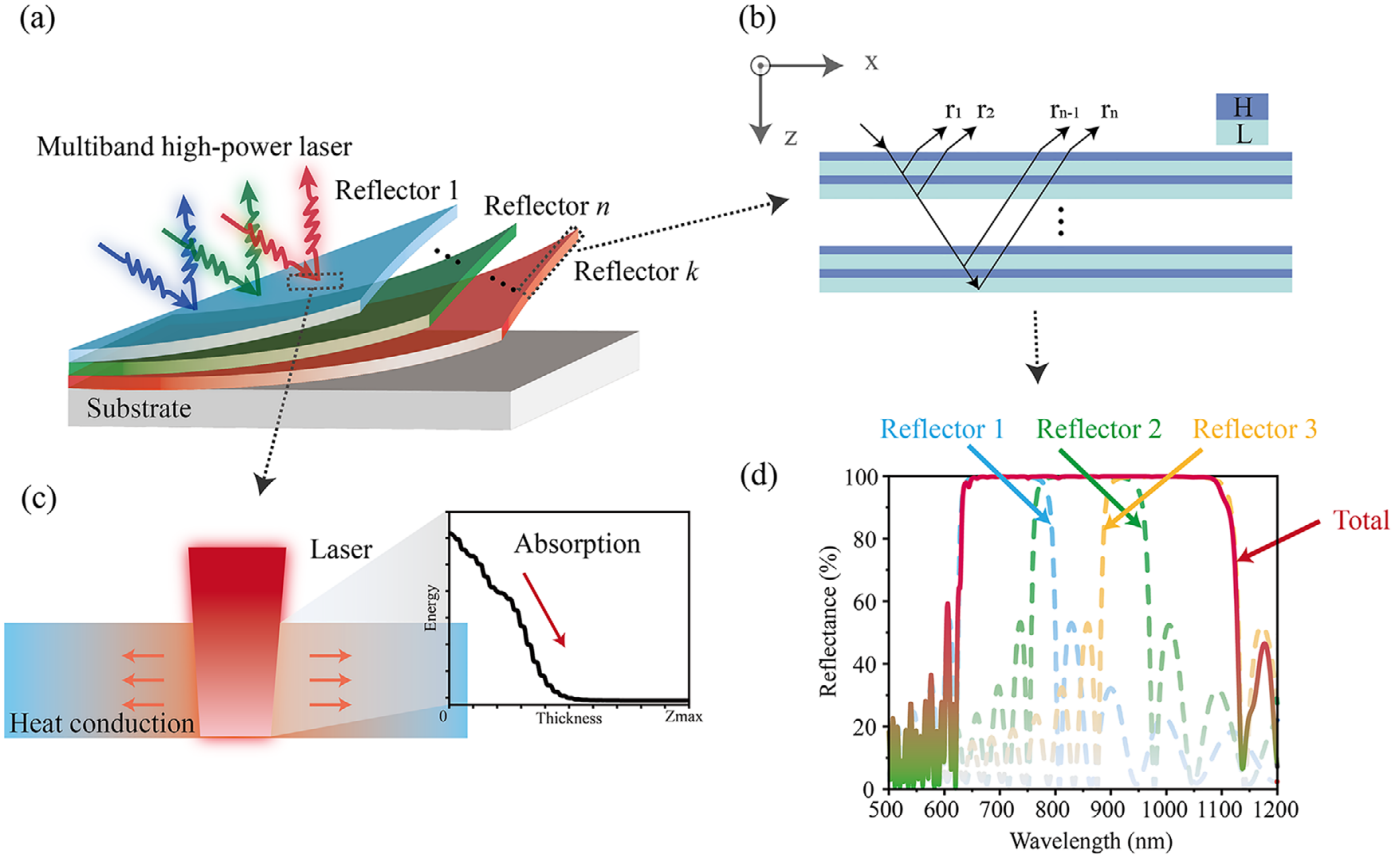
(a) Schematic of an ultrabroadband laser‐protection reflector, where multiple reflectors are stacked on the substrate. (b) Typical quarter‐wave stack structure of a reflector. (c) Temperature rise in the reflector due to the absorption of laser energy. (d) Reflectance spectra of three different reflectors and their total reflectance.

Multiband high‐power continuous‐wave lasers pose a significant threat to a wide range of targets owing to thermal effects and sustained energy deposition in optical and electronic systems. Simultaneous irradiation across multiple spectral bands can compromise conventional protective coatings, which are typically optimized for single‐wavelength defense. This drawback necessitates the development of advanced protection strategies to withstand multiband high‐energy laser exposure. To implement an ultrabroadband reflector, we designed a composite structure by stacking multiple reflectors (Figure 1a). Each reflector was realized using a typical nanometer quarter‐wave stack featuring alternating high‐ (H) and low‐refractive‐index (L) materials (Figure 1b), which provides high reflectance within the designed wavelength band. [40] However, because of the complex structure of the reflectors, their absorption behavior remains intricate and insufficiently understood (Figure 1c). Figure 1d shows that ultrabroadband reflection can be achieved by stacking three reflectors with central wavelengths of 700, 850, and 1000 nm (for reflectors 1, 2, and 3, respectively); each reflector possesses an A/(HL)^10/Sub nanostructure (where A represents the incident medium air, Sub represents the fused substrate, and H and L represent Ta_2_O_5_ and SiO_2_, respectively).

### 2.2 Multiband Laser‐Energy Distribution in Multilayer Films

**FIGURE 2:**
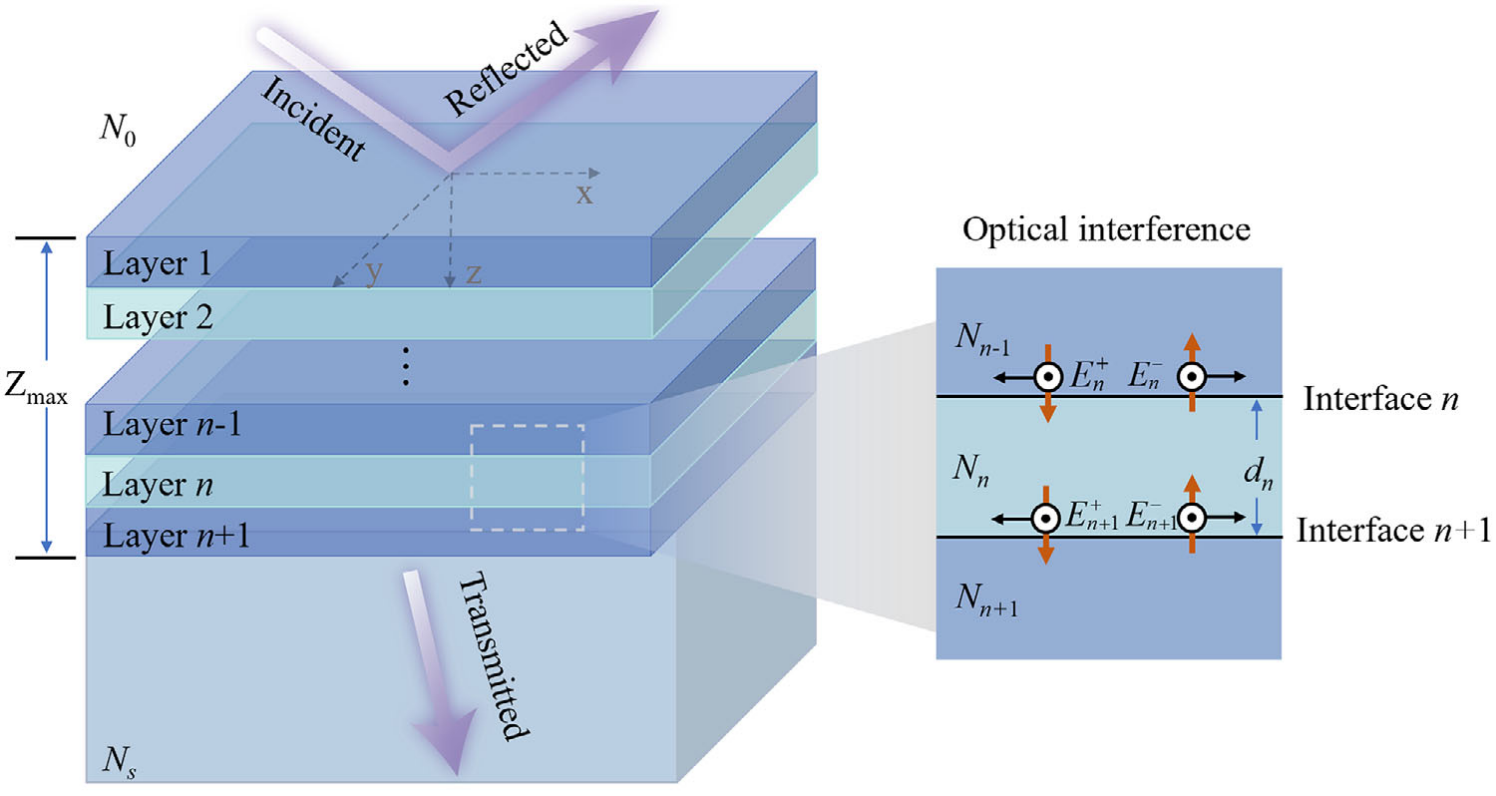
Schematic used to calculate the electric field and energy flow in a multilayer film.

The existence of an electric field within a film inevitably causes absorption of electromagnetic waves by the material of the film. To determine the laser‐energy distribution in multilayer films, we propose a novel laser‐energy absorption model for multilayer films that are subjected to multiband laser irradiation. Determining the electric‐field distribution within a multilayer structure penetrated by electromagnetic waves is necessary. Such waves are reflected at the interface between two media. Complex interference effects occur between the layers in a multilayer film (Figure 2). The electric field inside a film can be divided into forward‐propagating and backward‐propagating components. For a single‐layer film within the structure, the relationship between the electric and magnetic fields at interfaces *n* and *n* + 1 can be described using an optical transfer matrix:

(1) $$\left[\begin{array}{c} E_{n}\\ H_{n} \end{array}\right]=\left[\begin{array}{cc} \cos\delta_{n} & i\sin\delta_{n}/\zeta_{n}\\ i\zeta_{n}\sin\delta_{n} & \cos\delta_{n} \end{array}\right]\left[\begin{array}{c} E_{n+1}\\ H_{n+1} \end{array}\right],$$

where ζ_*n*_ represents the effective admittance; for s‐polarization, ζ_*n*_ = *N_n_* cos θ_*n*_, and for p‐polarization, ζ_*n*_ = *N_n_*/cos θ_*n*_; *N_n_* and *θ_n_* represent the refractive index of layer *n* and incident angle in layer *n*, respectively. Moreover, δ_*n*_ = 2π*N_n_d_n_* cos θ_*n*_/λ, where *d_n_* represents the thickness of layer *n* and *λ* represents the incident wavelength. Upon applying the boundary conditions, the tangential components of the electric and magnetic fields become continuous across the interface between adjacent materials:

(2) $$\left\{\begin{array}{c} n\times (E_{n}-E_{n+1})=0\\ n\times (H_{n}-H_{n+1})=0 \end{array}\right.,$$

where **n** denotes the normal vector of the film surface. The electric‐field amplitude distribution in a nano‐multilayer film can then be expressed as follows:

(3) $$\left[\begin{array}{c} E_{0}\\ H_{0} \end{array}\right]=\prod_{n=1}^{k}\left\{\left[\begin{array}{cc} \cos\delta_{n} & i\sin\delta_{n}/\zeta_{n}\\ i\zeta_{n}\sin\delta_{n} & \cos\delta_{n} \end{array}\right]\right\}\left[\begin{array}{c} E_{k+1}\\ H_{k+1} \end{array}\right].$$

Because the electric field exhibits different intensities at different positions within the film, the multilayer structure can be discretized into multiple sublayers, and the electric‐field distribution can be obtained using **Equation** **3**. The normal component of the electromagnetic field can be obtained as follows:

(4) H(z)=k×E(z),

where **k** is the wave vector. Applying the Poynting theorem, the average amount of laser energy per unit area that crosses the plane perpendicular to the Z axis can be expressed as follows:

(5) $$P\left(z\right)=\frac{1}{2}Re\left(E\left(z\right)\times H^{*}\left(z\right)\right).$$

The derivation describes the energy distribution within the film for a monochromatic wave, whereas the spatial energy distribution under multiband laser irradiation can be obtained by integrating the incident spectral intensity over the broadband spectrum:

(6) $$\mathrm{IntegralP}\left(z\right)=\int P(\lambda ,z)\cdot Spectrum(\lambda )d\lambda 0\leq z\leq Z_{\max}.$$

The wavelength‐dependent P(*λ*, *z*) parameter can be determined from **Equation** **5**. *Spectrum* refers to the spectral intensity distribution of the incident multiband laser, and *Z*_max_ represents the total film thickness.

### 2.3 Design and Characterization of Ultrabroadband Reflective Coatings

As shown in Figure 1 d, each reflector corresponds to a different reflection band; therefore, the stacking order of the reflectors determines the sequence in which the different spectral regions are reflected. If the short‐wavelength reflector is placed close to the incident medium (air), then short‐wavelength light is reflected first. As discussed in Section 2.2, different multilayer structures affect the electric‐field distribution within the coatings, and this distribution in turn influences the temperature rise in the structures. Therefore, the final design of the ultrabroadband reflector determines this rise under broadband laser irradiation. Three broadband high‐reflectivity mirrors (BRMs), with an average reflection bandwidth of 450–1250 nm and a theoretical average reflectance greater than 99.9%, are designed. For BRM1, a quarter‐wave stack with short‐wavelength‐prioritized reflection is employed. For BRM2, a long‐wavelength‐prioritized chirped‐layer structure was adopted to achieve high reflectance with a relatively small number of layers and to reduce the overall thickness. For BRM3, a quarter‐wave stack with long‐wavelength‐prioritized reflection is used. The ultrabroadband protective design overcomes the limitations of single‐wavelength coatings, which fail at large incidence angles.

**FIGURE 3:**
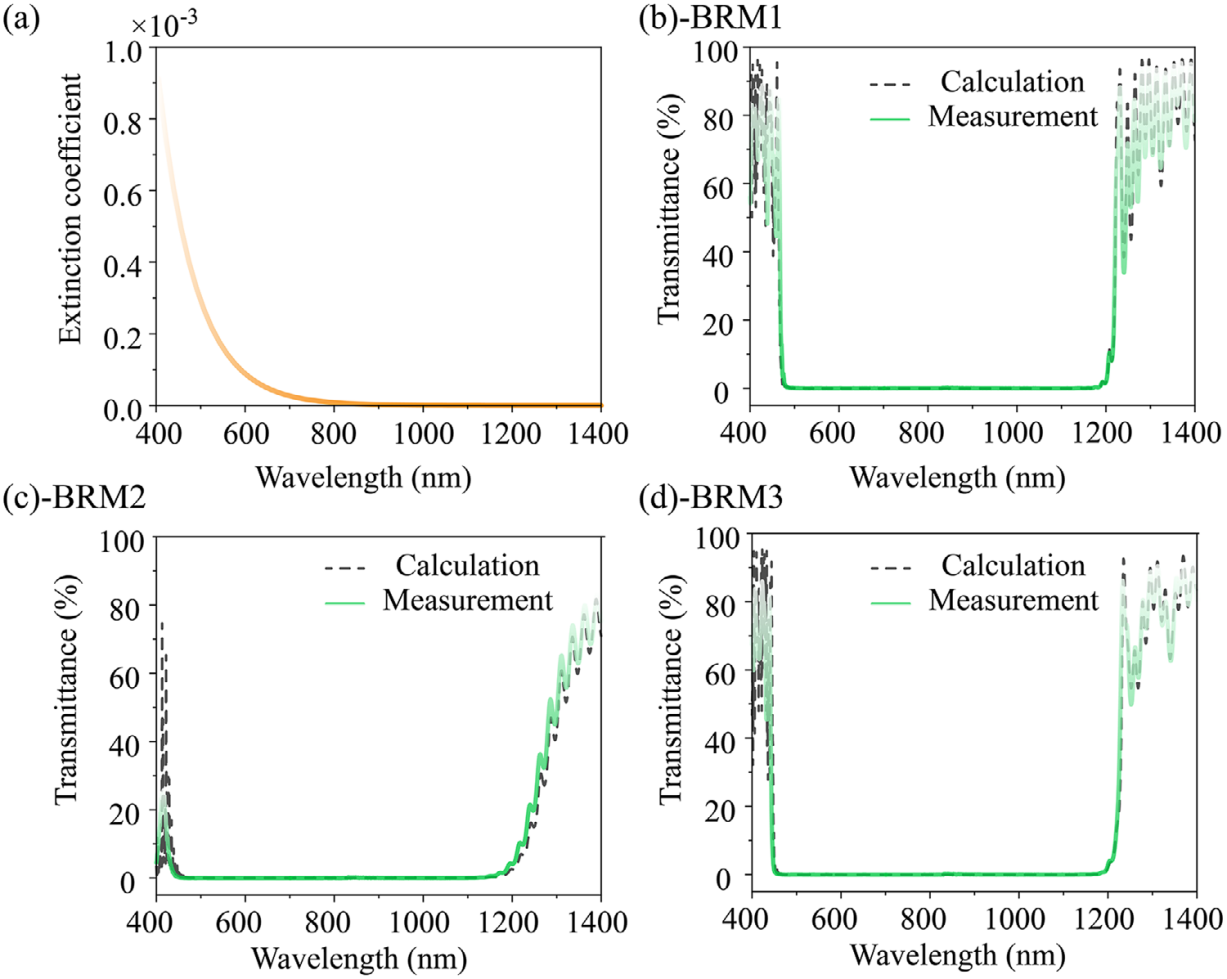
(a) Extinction coefficient of Ta_2_O_5_; (b–d) Theoretical and measured transmittances of BRM1, BRM2, and BRM3.

The bandwidth of the reflection band at the design wavelength is significantly affected by the refractive index ratio of the quarter‐wave stack. The following equation provides the half‐width of the reflection band under normal incidence:

(7) $$\Delta g=\frac{2}{\pi }\arcsin\left(\frac{n_{H}-n_{L}}{n_{H}+n_{L}}\right),$$

whereΔ*g*represents the half‐width of the reflection band, and*n_H_*,*n_L_* represent the refractive indices of H and L in the quarter‐wave stack, respectively. **Equation** **7** indicates that a high *n_H_*,*n_L_* ratio results in a broad high‐reflection band. Therefore, we selected Ta_2_O_5_ (among the commonly used optical materials, Ta_2_O_5_ offers a relatively high refractive index) and SiO_2_ as the H and L materials, respectively. The refractive indices of the H and L materials are described using the Cauchy formula (**Equation** **8**) obtained from commercial OptiChar, with the corresponding coefficients listed in **Table** **1**:

(8) $$n\left(\lambda \right)=A_{0}+A_{1}/\lambda^{2}+A_{2}/\lambda^{4},$$

where *λ* is the wavelength (µm) and *A*_0_, *A*_1_, and *A*_2_ are the Cauchy coefficients. Because silica exhibits extremely low absorption, only the absorption of Ta_2_O_5_ is considered; its extinction coefficient, shown in Figure 3 a, is significantly higher at shorter wavelengths than at longer wavelengths. Figure 3b–d shows that the measured results for the three designs are consistent with the intended results. In addition, the three designs exhibit an experimentally measured transmittance of less than 0.1% in the 450–1200 nm range. We further quantified the absorption and scattering losses of the coatings. Weak absorption at 532 nm and 1064 nm was measured using a self‐developed surface thermal lens (STL) technique. The scattering loss was evaluated from atomic force microscopy (AFM, measured by Dimension‐3100) measurements using the classical relation between RMS roughness and total integrated scattering (TIS). The combined loss budget—absorption, TIS‐derived scattering, and measured transmittance less than 0.01% at both wavelengths—indicates that all three multilayer reflectors achieve reflectivity greater than 99.7% at 532 nm and 99.9% at 1064 nm. Detailed data are provided in Section S1. The final thickness of both BRM1 and BRM3 is approximately 25 µm, while that of BRM2 is approximately 19 µm. All the samples were fabricated on fused silica substrates with a diameter of 50 mm and a thickness of 5 mm. The design parameters for the three reflectors are provided in Section S1.

**TABLE 1:** Cauchy parameters of the materials selected in this study.

Materials	*A*_0_	*A*_1_	*A*_2_
Ta_2_O_5_	2.01485	0.0301196	−0.000763967
SiO_2_	1.44293	0.0116192	−0.000370928

**FIGURE 4:**
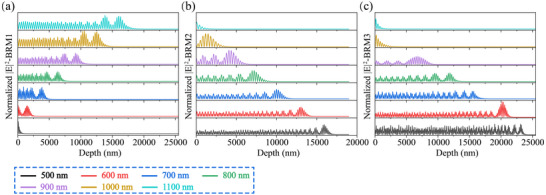
(a–c) Electric‐field distributions of BRM1, BRM2, and BRM3 at 100‐nm intervals within the 500–1100 nm wavelength range.

Equation 3 yields the electric‐field distributions inside the films of the three designed reflectors. Figure 4 a–c presents the electric‐field distributions at 100‐nm intervals within the 500–1100 nm wavelength range for BRM1, BRM2, and BRM3. Evidently, the penetration depth varies with wavelength and design. For BRM1, which is designed to preferentially reflect short wavelengths, the electric‐field penetration depth at shorter wavelengths is extremely shallow; however, it increases with the wavelength. Both BRM2 and BRM3 prioritize long‐wavelength reflection. Therefore, their short‐wavelength components penetrate deeper than those of BRM1. In contrast, the penetration depth decreases upon increasing the incident wavelength. The BRM2 adopts a nonperiodic structural design; therefore, the electric‐field penetration depths at various wavelengths are generally slightly smaller than those of BRM3.

### 2.4 Temperature Rise in Ultrabroadband Reflectors Under SC Laser Illumination

**FIGURE 5:**
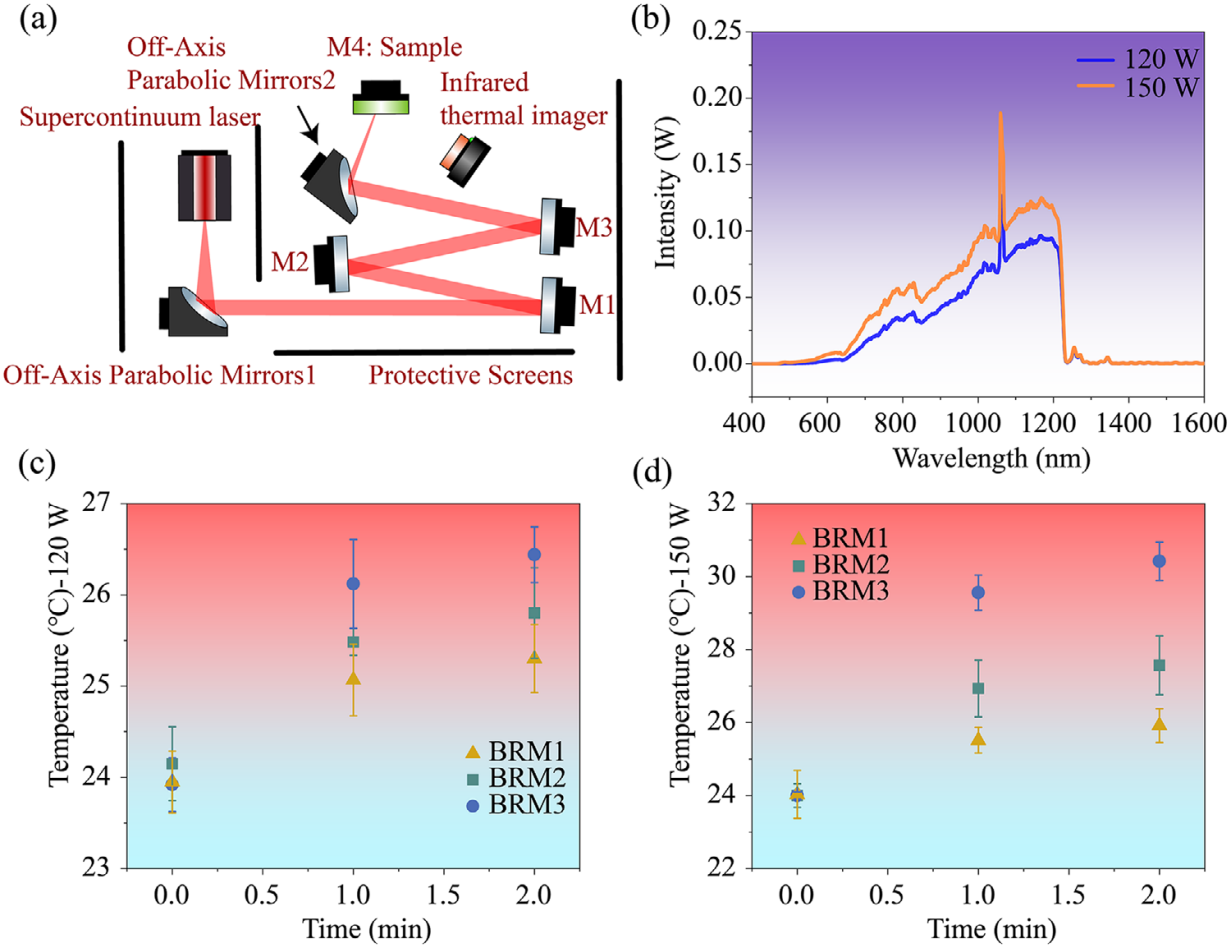
(a) Setup for temperature‐rise testing under SC laser irradiation. (b) Spectral intensity distributions after reflection by a parabolic mirror, following filtering by the mirrors, at output powers of 120 and 150 W. (c,d) Surface temperature of the three mirror designs after 1 and 2 min of irradiation under laser‐output powers of 120 and 150 W. For each design, five independent samples were tested (*n* = 5); the results are presented as mean ± standard deviation (SD).

Owing to the broad spectral coverage of the SC laser from short to long wavelengths, the three film designs exhibit distinct energy‐absorption behaviors that are attributed to their structural differences. Accordingly, as shown in Figure 5a, an experimental platform was constructed to measure the temperature rise in each design under SC laser irradiation. A custom‐built continuous‐wave SC laser, with a maximum output power of 150 W, was used. The laser beam emitted from the fiber end cap was first collimated by an off‐axis parabolic mirror (PM1, RFL (reflective focal length) = 38.1 mm), and then reflected and spectrally filtered at small incidence angles by three BRM3. It was subsequently focused using a second off‐axis parabolic mirror (PM2, RFL = 76.2 mm) before being directed onto the sample surface. The resulting beam had a spot diameter of approximately 8 mm. An infrared thermal camera was employed to monitor the maximum surface temperature rise in the sample in real time. The changes in temperature were recorded after 1 and 2 min of laser irradiation. Five samples of each design were prepared for these experiments.

**FIGURE 6:**
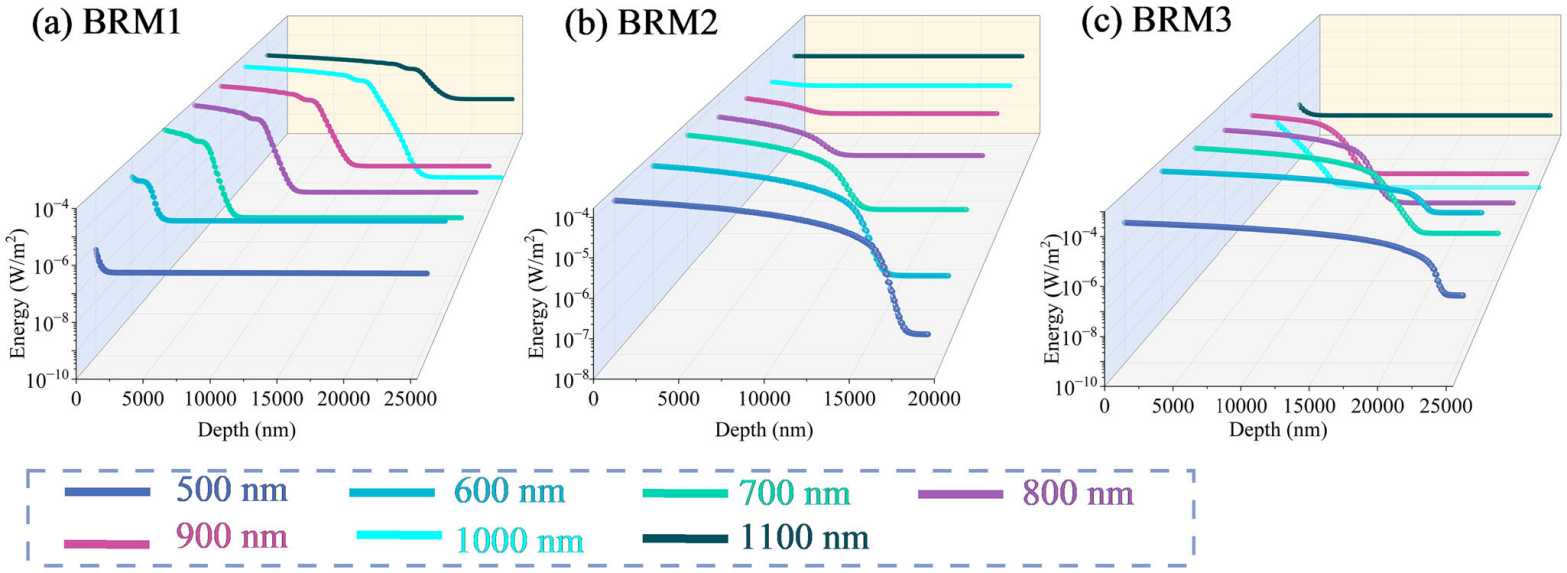
(a–c) Laser‐energy absorption distributions for BRM1, BRM2, and BRM3 at different wavelengths.

The spectrum measured by the spectrometer after the beam was reflected by the three BRM3 mirrors is shown in Figure 5b. The results indicate that wavelengths greater than approximately 1300 nm were effectively filtered, and the spectral shape remained stable irrespective of the laser's output power. The overall increase in spectral intensity was greater at high output powers than at low power values; specifically, the relative enhancement in the short‐wavelength region was more pronounced than that in the long‐wavelength region. Moreover, a distinct seed signal was observed at 1064 nm. Figure 5c,d shows the maximum surface temperatures of the samples, measured by the infrared thermal camera, for the three designs under laser‐output powers of 120 and 150 W at the 1‐ and 2‐min time points, respectively. The temperature rise for the three samples was noticeably smaller under low‐power (120 W) laser irradiation than under high‐power (150 W) laser irradiation, with BRM1 exhibiting the smallest temperature rise. In addition, under irradiation at the same power, BRM1, which prioritizes short‐wavelength reflection, shows the least temperature rise, whereas BRM3, which prioritizes long‐wavelength reflection, exhibits the greatest temperature rise. When the laser output power was increased to 150 W, the temperatures of all the samples increased, with BRM3 exhibiting the greatest rise of approximately 6.5°C. Under both irradiation powers, the temperature rise in BRM2 was relatively smaller than that in BRM3. This phenomenon can be explained using the model described in Section 2.2. The spectrally filtered laser, with the intensity distribution shown in Figure 5b, was used as the incident source, and the laser‐energy absorption model described in Section 2.2 was applied to calculate the actual energy distributions for BRM1, BRM2, and BRM3 under laser output powers of 120 and 150 W. The corresponding energy distributions at different wavelengths are shown in Figure 6a–c. A comparison of the results obtained for BRM1 and BRM3, which differ only in the order of their reflection bands, demonstrates that deeper field penetration causes greater laser energy absorption.

**FIGURE 7:**
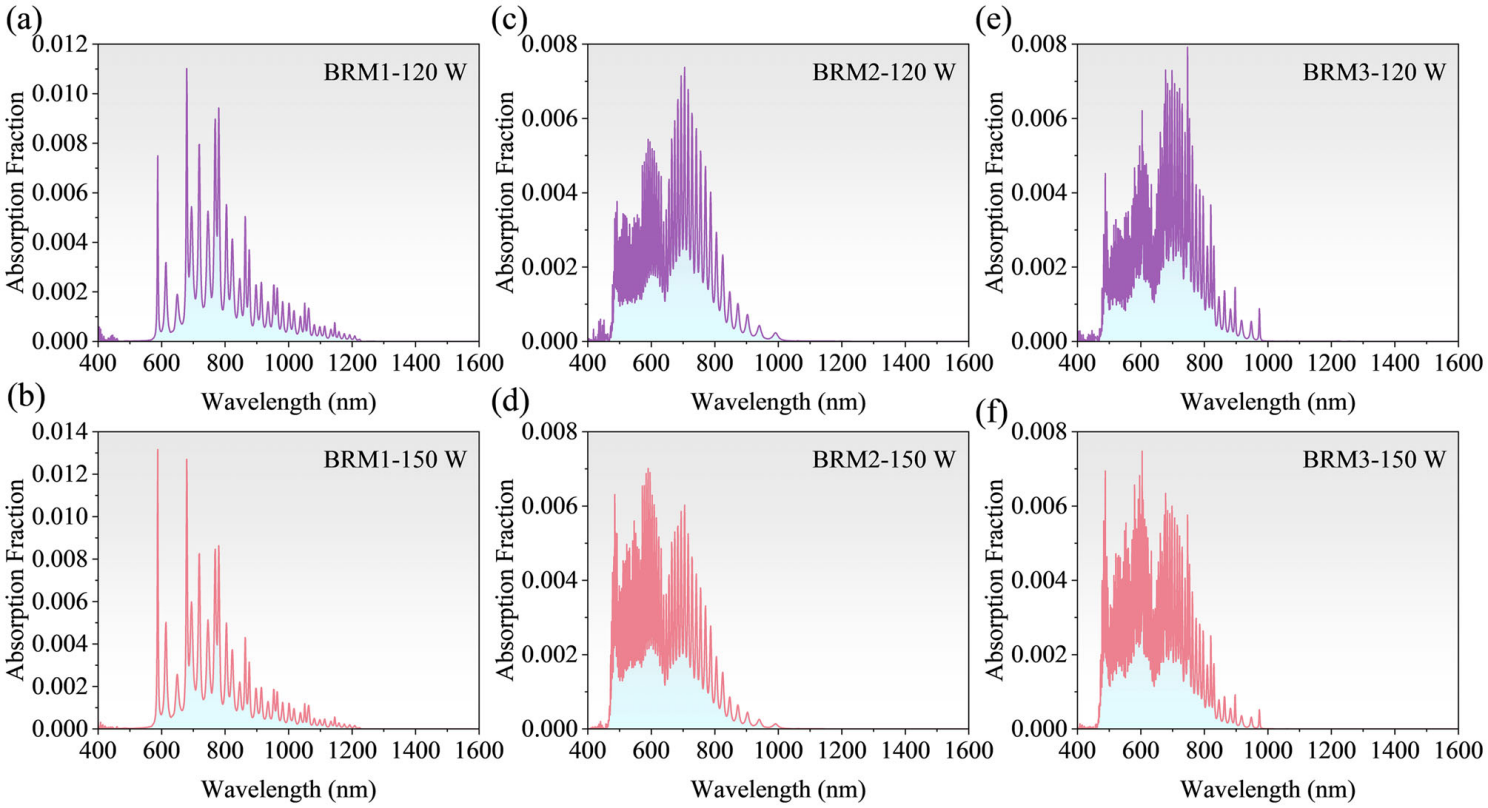
(a–b), (c–d), and (e–f) show the wavelength‐dependent fractional absorption for BRM1, BRM2, and BRM3 under incident powers of 120 and 150 W, respectively. In each plot, the absorption at every wavelength is normalized by the total absorbed energy, representing the fractional contribution of each spectral component to the overall absorption.

Moreover, the extinction coefficient of Ta_2_O_5_ is significantly higher at shorter wavelengths than at longer wavelengths (Figure 3a). Therefore, because BRM3 allows deeper short‐wavelength penetration than BRM1, the overall energy dissipation by the former is expected to be higher than that of the latter. For BRM2, which features an aperiodic structure that preferentially reflects long wavelengths, the overall field penetration is slightly shallower than that of BRM3, resulting in lower energy absorption. Although the intensity of the long‐wavelength region of the SC spectrum is significantly higher than that of the short‐wavelength region, the strong material absorption at short wavelengths results in significant heating (Figure 5b). Consequently, even dielectric mirrors designed to preferentially reflect long wavelengths cannot effectively suppress the temperature rise. Among the three designs, BRM1, which prioritizes short‐wavelength reflection, achieves the least temperature rise. Moreover, the spectral intensity at 150 W was higher than that at 150 W. Nevertheless, only BRM1, which preferentially reflects short wavelengths, exhibits a relatively smaller temperature rise. To further explain this, we quantitatively evaluated the wavelength‐dependent absorption. Specifically, the absorption at each wavelength (calculated using Equation 5) was first summed over the entire spectral range to obtain the total absorbed energy. Then, the fractional contribution of each wavelength was determined by dividing its absorption by the total value. The resulting absorbed‐energy distribution is presented in Figure 7. The results clearly show that, the short‐wavelength regions of the three designs consistently provide the dominant contribution to the total absorbed energy. This trend appears under both irradiation conditions examined in our study, namely incident powers of 120 and 150 W. Moreover, when the incident power was increased to 150 W, the short‐wavelength portion of the spectrum was enhanced accordingly, thereby further amplifying short‐wave absorption for the three designs. These observations establish a solid quantitative basis to conclude that short‐wave components govern the heating behavior.

### 2.5 Finite‐Element Simulation of Temperature Rise in Broadband Reflector

Figure 6 shows the laser‐energy absorption behavior within the films at each wavelength for the three mirror designs. To calculate the temperatures of BRM1, BRM2, and BRM3 subjected to SC laser irradiation, the energy‐absorption contributions from all the spectral components were integrated over the incident spectrum using **Equation** **6**.

**FIGURE 8:**
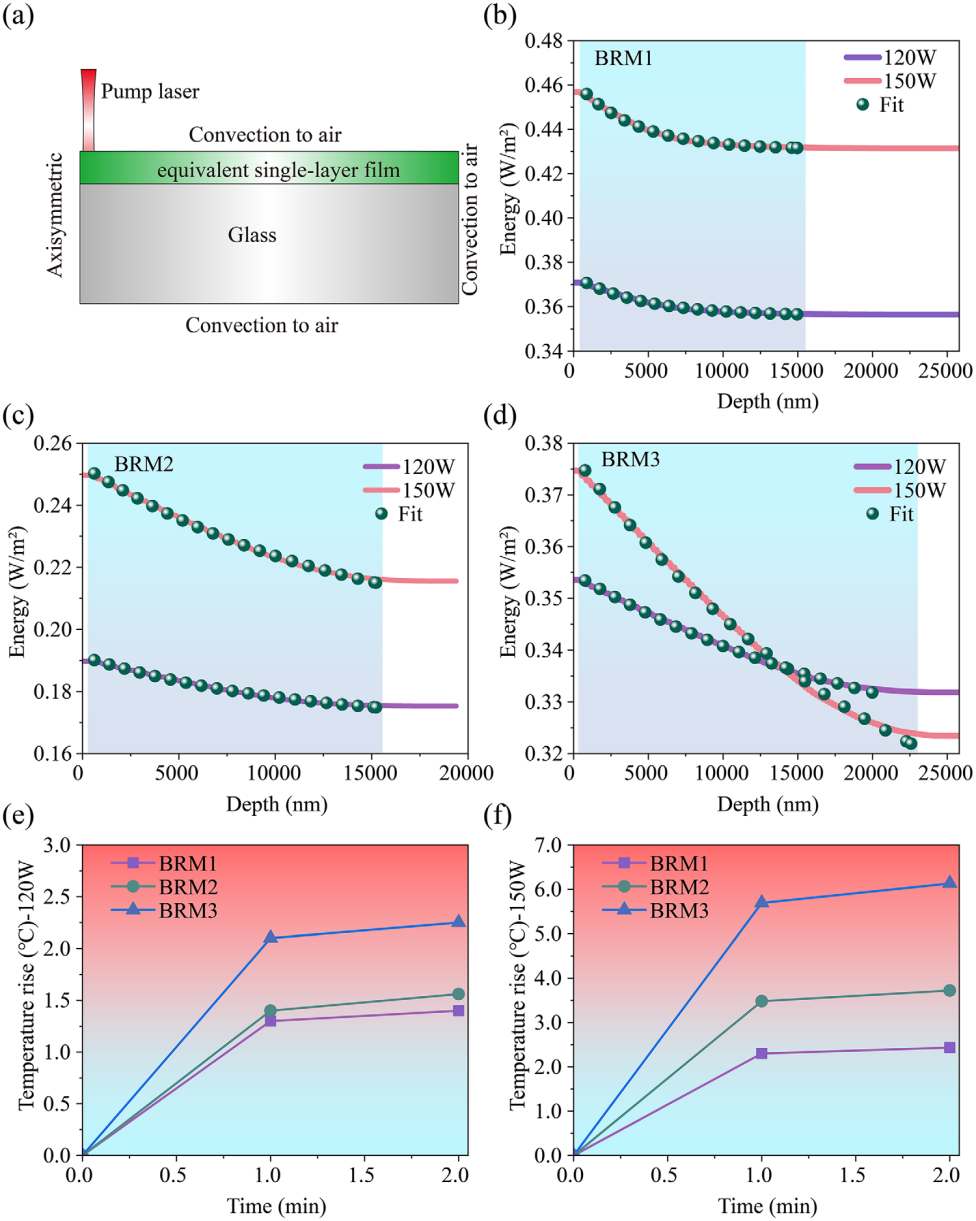
(a) Schematic of the finite‐element model for temperature‐rise calculation; (b–d) Energy‐absorption behaviors of BRM1, BRM2, and BRM3 under laser irradiation at 120 and 150 W. (e,f) Simulated temperature rise for the three BRM designs after 1 and 2 min of irradiation at 120 and 150 W.

As shown in Figure 8a, the multilayer stack was treated as an equivalent single layer because the thermal‐diffusion length under second‐level irradiation was two orders of magnitude larger than the coating thickness, making the coating effectively isothermal. Additional comparative analyses confirming the validity of this simplification are provided in Section S2. The integrated broadband laser energy decreases within the multilayer film, indicating that a fraction of the energy is absorbed by the film. For the three designs, the overall energy absorption within the film exhibits an approximately exponential decay distribution. Figure 8b–d shows that the energy‐absorption behaviors of the three designs vary as BRM1< BRM2< BRM3. An exponential decay function, such as the one in **Equation** **9**, can be used to fit the energy‐absorption data. Figure 8b–d indicates excellent agreement between the fitted curves and the results calculated at 120 and 150 W. Detailed fitting parameters are provided in Section S2.

A 2D axisymmetric heat conduction equation is used to calculate the temperature in this simplified model. The laser source exhibits a Gaussian distribution, and the intensity term *I* is expressed by **Equation** **10** (where *P* represents the laser power, and *w*(*z*_0_) denotes the beam‐waist radius at the sample surface).

(9) $$\varepsilon \left(z\right)=a\exp\left(-z/b\right)+\varepsilon_{0},$$

(10) $$I\left(r,z\right)=\frac{2P}{\pi \omega {\left(z_{0}\right)}^{2}}\exp\left(\frac{-2r^{2}}{\omega {\left(z_{0}\right)}^{2}}\right)\varepsilon \left(z\right),$$

(11) $$\rho c\frac{\partial T}{\partial t}=\nabla \cdot \left(k\nabla T\right)+Q\left(r,z\right),$$

(12) $$\begin{array}{c} Q\left(r,z\right)=-\frac{d}{dz}I\left(r,z\right)=\left\{\frac{2P}{\pi \omega {\left(z_{0}\right)}^{2}}\exp\left(\frac{-2r^{2}}{\omega {\left(z_{0}\right)}^{2}}\right)\right\}\alpha_{1}\exp\left(-z\alpha_{2}\right)\\ \alpha_{1}=\frac{a}{b},\alpha_{2}=\frac{1}{b} \end{array}.$$

In **Equation** **11**, which represents heat conduction, *ρ* and *c* denote the material density and specific heat capacity, respectively; *T* denotes temperature; and *k* is the thermal conductivity. Because the sample is cylindrical, the Laplacian operator is expressed in axisymmetric cylindrical coordinates The second term, *Q*(*r*, *z*), on the right‐hand side of the equation represents the heat source. Because the energy absorption corresponds to the heat source term *Q*(*r*, *z*), **Equation** **12** can be derived, with the coefficients *α*_1_ and *α*_2_ precisely describing the energy attenuation within the multilayer film. This set of equations provides a simple and effective model for describing the multiband‐laser‐propagation and absorption behaviors in multilayer films. Because of the minimal laser penetration into the substrate, the absorption in the substrate is significantly lower than that in the film. Therefore, only the absorption within the film layer is considered. The COMSOL simulation was used to calculate the temperature rise in the films. The simulation details are provided in Section S2. Figure 8e,f depicts the simulated temperature‐rise behaviors of the three designs, demonstrating excellent agreement between simulated and experimental results at both laser output powers of 120 and 150 W.

## 3 Conclusion

In this study, a novel energy‐absorption model, based on spectral intensity integration, was proposed to describe the absorption behavior of multilayer dielectric films irradiated by an SC laser. Calculations revealed that absorption in the short‐wavelength region contributed more significantly to the temperature rise than did absorption of the material in the long‐wavelength region. This result could be primarily attributed to the higher absorption shown by the material at shorter wavelengths, even when the long‐wavelength intensity of the SC laser was higher than its short‐wavelength counterpart. Three broadband laser‐protective reflector designs with different structures were developed, and their temperature‐rise behaviors under SC laser irradiation were investigated. The BRM1 employed a nanoquarter‐wave stack designed to preferentially reflect short wavelengths. The BRM2 adopted a long‐wavelength‐prioritized structure with chirped layer thickness. While the design approach of BRM3 was the same as that of BRM1, the former prioritized long‐wavelength reflection. A temperature‐rise testing platform was developed using an SC laser, and the three designed reflectors were exposed to the same spectral intensity at different output powers. The experimental results showed that BRM1, i.e., the short‐wavelength‐reflecting design, exhibited the smallest temperature rise (below 2.5°C) in temperature at both output powers of 120 and 150 W. In contrast, BRM3, which possessed a similar structure but prioritized the reflection of long wavelengths, showed the greatest temperature rise of approximately 6.5°C at an output power of 150 W. The BRM2, with its chirped structure, exhibited an intermediate temperature rise at both the output powers. An exponentially decaying laser‐energy distribution was observed within the multilayer films. Two absorption coefficients were employed to precisely describe the energy attenuation within the multilayer film. Based on the proposed model, the multilayer structure was reduced to an equivalent single‐layer film to efficiently simulate the temperature rise via finite‐element analyses. The simulation results were in excellent agreement with the experimental measurements.

These results reveal a non‐intuitive but important thermal‐design principle for broadband laser‐protective mirrors: the priority of the reflection band cannot be optimized solely based on optical reflectance; instead, it must be determined by evaluating heat deposition resolved across the spectrum. Furthermore, the proposed model can be extended to arbitrary multilayer structures and material systems (including metallic multilayers), and it is in principle applicable to simulate nanosecond pulses. This provides a powerful and general framework for the thermal evaluation and design of broadband, multiband, and multiwavelength laser‐protective dielectric coatings.

## 4 Experimental Details

### 4.1 Fabrication

Ta_2_O_5_/SiO_2_ multilayer films were fabricated by dual‐ion beam sputtering (Veeco Instruments Inc., USA). During the deposition process, which was conducted at a beam current of 500 mA and bias voltage of 1200 V, the vacuum level was maintained at 2.0 × 10^−^^6^ Torr. Oxygen and argon gases were used during the process.

### 4.2 Characterization

The transmittances of the films were measured using an ultraviolet–visible–near‐infrared spectrophotometer (Lambda 1050). The laser power was measured using an Ophir 250 W power meter. The SC laser spectral distribution was recorded using a YOKOGAWA AQ6374 optical spectrum analyzer (350–1750 nm, 1200–2400 nm). In addition, an infrared thermal imager (Fluke Ti480 Thermal) was integrated in the setup for sample analysis. Weak absorption at 532 and 1064 nm was measured using a self‐developed Surface Thermal Lens (STL) technique. The RMS roughness was measured using atomic force microscopy (AFM, measured by Dimension‐3100). The van der Waals force between atoms was tested to determine the surface characteristics of the sample; each test point was a rectangular area of 5 × 5 µm^2^.

### 4.3 Statistical Analysis

For each multilayer design (BRM1–BRM3), temperature‐rise measurements were performed using five independent samples (*n* = 5); the results are reported as mean ± SD. Weak absorption at 532 and 1064 nm was measured using two samples per design, with 10 measurement points on each sample (*n* = 20 measurements per design in total). The absorption data were averaged and reported as mean ± SD. The AFM surface roughness was measured using two samples per design, with three measurement points on each sample (*n* = 6 measurements per design). The roughness values are reported as mean ± SD. No hypothesis testing or significance analysis was performed. All statistical processing and plotting were performed using OriginPro 2025.

## Author Contributions

Yu‐Kang Feng: Conceptualization, Investigation, Data curation, Writing – original draft, preparation. Yan‐Zhi Wang: Supervision. Yu‐lin Zhang: Writing – review & editing. Ye‐Sheng Lu: Writing – review & editing. Yu Chen: Supervision. Fan‐xin Meng: Writing – review & editing. Hui‐song Hu: Writing – review & editing. Zhong‐Yang Xing: Writing – review & editing. Jian‐Da Shao: Funding acquisition.

## Funding

This work was supported by the National Natural Science Foundation of China (62405340), Shanghai Pujiang Program (24PJD127), Open Project Program of Wuhan National Laboratory for Optoelectronics (2024WNLOKF008), National Key R&D Program of China (2022YFB3605903), China Postdoctoral Science Foundation (2022M723268), International Partnership Program of the Chinese Academy of Sciences (181231KYSB20200040), Youth Innovation Promotion Association of the Chinese Academy of Sciences (Y2021072), and Strategic Priority Research Program of CAS (XDB1603).

## Conflicts of Interest

The authors declare no conflicts of interest.

## Supporting information




**Supporting File**: advs74067‐sup‐0001‐SuppMat.docx

## Data Availability

The data that support the findings of this study are available from the corresponding author upon reasonable request.;
