# Supplementary material for: Spectral‐Integrated Thermal Absorption Model for Broadband Laser‐Protective Reflectors Under Supercontinuum Irradiation
Source: Adv Sci (Weinh). 2026 Feb 15;13(19):e18750. doi: 10.1002/advs.202518750 (PMC13045233; doi:10.1002/advs.202518750)
Supplement: Supplementary file 1 — Supporting File: advs74067‐sup‐0001‐SuppMat.docx [file ADVS-13-e18750-s001.docx]

Supporting Information

**Spectral-Integrated Thermal Absorption Model for Broadband Laser-Protective Reflectors under Supercontinuum Irradiation**

Yukang Feng, Yanzhi Wang, * Yulin Zhang, Yesheng Lu, Yu Chen, * Fanxin Meng, Huisong Hu, Zhongyang Xing, and Jianda Shao *

*yanzhiwang@siom.ac.cn; chenyu4@siom.ac.cn; jdshao@siom.ac.cn.

Section 1 Design information of the three BRM samples


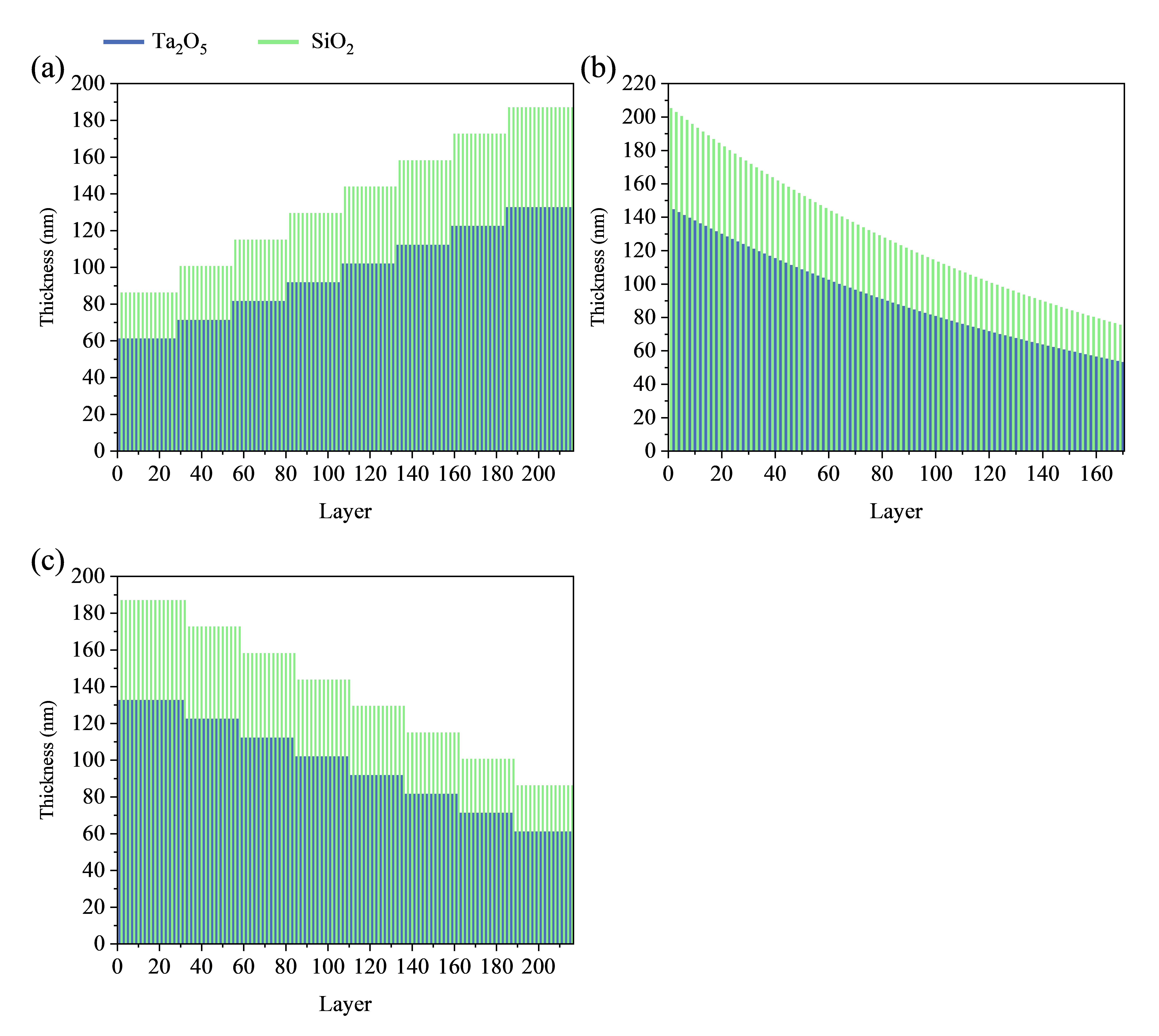


**Figure S1** (a)–(c) Film thicknesses of BRM1, BRM2, and BRM3.

**Figure S1** (a–c) show the film thicknesses of BRM1, BRM2, and BRM3. Layer numbering starts from the side closest to air, with the first layer labeled as Layer 1. As shown in Figure S1 (a) and (c), the layer-thickness distributions of BRM1 and BRM3 are reversed.

Detailed absorption and scattering data for all three reflectors:

We experimentally measured the absorption loss at two representative wavelengths, 532 and 1064 nm, for all three designs. Two coated samples were selected for each design, and ten measurement points were recorded on each sample using a self-developed surface thermal lens (STL) technique. During the measurements, the data at 532 nm showed instability and larger fluctuations compared with the results at 1064 nm. The final measured values are summarized in Table S1. To evaluate the scattering loss, we characterized the surface roughness of the coatings using atomic force microscope (AFM), which was measured by Dimension-3100. The classical relation between surface roughness and total integrated scattering (TIS) ^[1]^ is as follows:

$TIS=\left(\frac{4\pi\sigma\cos\theta}{\lambda}\right)^{2}$ 1

where *σ* is the RMS roughness and *λ* is the wavelength; as show in **Equation 1** the corresponding scattering loss is on the order of at 532 and 1064 nm for the measured roughness values. For each design, the RMS roughness was measured at six points (two samples, three points per sample) and averaged. The TIS at 532 and 1064 nm under *θ* = 0 was then calculated from the averaged RMS roughness. From the absorption data, it can be observed that BRM1 exhibits lower weak absorption at 532 nm compared with BRM2 and BRM3, while its absorption in the long-wavelength region is higher. Combined with the TIS results and the measured transmittance (< 0.01% both at 532 and 1064 nm), it is inferred that the three designs achieve reflectivity greater than 99.7% at 532 nm and greater than 99.9% at 1064 nm.

**Table S1** Measured absorption loss values at 532 and 1064 nm.

| Design | Sample# | Absorption @532 nm | | Absorption @1064 nm | |
| --- | --- | --- | --- | --- | --- |
|  |  | Mean ± SD (ppm) | Max (ppm) | Mean ± SD (ppm) | Max (ppm) |
| BRM1 | 1 | 226.79 ± 48.03 | 282.50 | 540.90 ± 16.02 | 556.70 |
| BRM1 | 2 | 198.00 ± 52.35 | 255.60 | 555.53 ± 16.66 | 578.90 |
| BRM2 | 1 | 1573.18 ± 314.13 | 1913.90 | 37.97 ± 1.74 | 40.00 |
| BRM2 | 2 | 1656.33 ± 250.79 | 1941.10 | 55.01 ± 0.65 | 56.10 |
| BRM3 | 1 | 1712.16 ± 103.20 | 1873.10 | 4.38 ± 1.28 | 7.00 |
| BRM3 | 2 | 1082.17 ± 285.98 | 1537.50 | 54.85 ± 3.67 | 60.90 |

**Table S2** Measured RMS and corresponding TIS values (calculated using the averaged RMS) at 532 and 1064 nm.

| Design | Sample# | Point1  RMS (nm) | Point2  RMS (nm) | Point3  RMS (nm) | Mean ± SD RMS (nm) | TIS  @532 nm | TIS  @1064 nm |
| --- | --- | --- | --- | --- | --- | --- | --- |
| BRM1 | 1 | 0.576 | 0.505 | 0.550 | 0.544 ± 0.036 | 1.65E-4 | 4.12E-5 |
| BRM1 | 2 | 0.555 | 0.491 | 0.554 | 0.533 ± 0.037 | 1.59E-4 | 3.97E-5 |
| BRM2 | 1 | 0.544 | 0.473 | 0.548 | 0.522 ± 0.042 | 1.52E-4 | 3.80E-5 |
| BRM2 | 2 | 0.480 | 0.477 | 0.518 | 0.492 ± 0.023 | 1.35E-4 | 3.37E-5 |
| BRM3 | 1 | 0.499 | 0.511 | 0.575 | 0.528 ± 0.041 | 1.56E-4 | 3.89E-5 |
| BRM3 | 2 | 0.537 | 0.471 | 0.463 | 0.490 ± 0.041 | 1.34E-04 | 3.35E-05 |


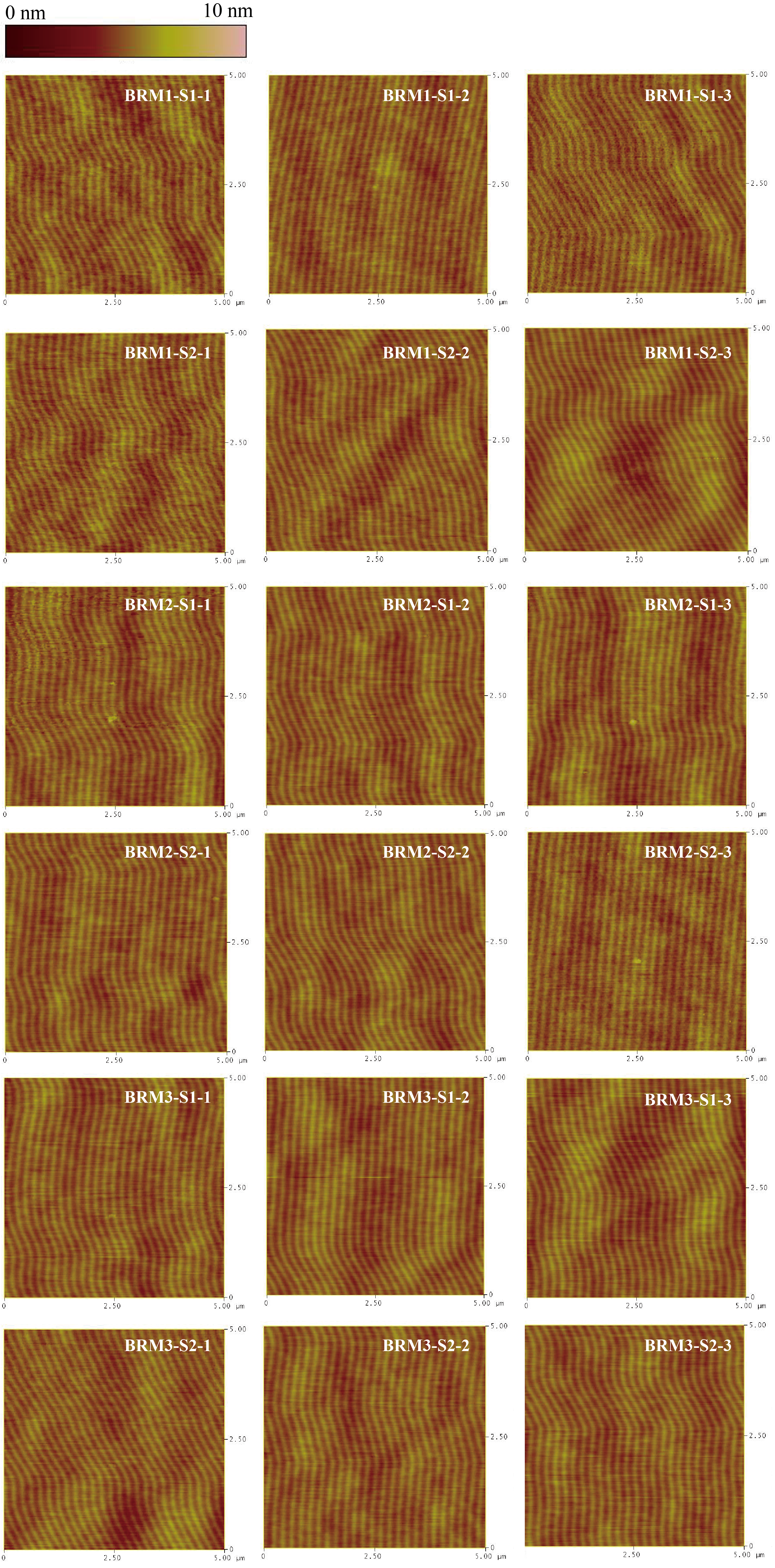


**Figure S2** Two-dimensional AFM images (measured by Dimension-3100) of the BRM1-3.

Section 2. Temperature-rise simulation using COMSOL

Sensitivity of the heat-conduction solution to material parameters

The thermal diffusivity *α* ^[2]^ (**Equation 2**) of for typical oxide materials, such as SiO_2_ is approximately 8.7×10^-7^ m^2^/s. Therefore, the characteristic thermal diffusion length ranges from several hundred micrometers to approximately one millimeter for irradiation times on the order of s. This diffusion length is two orders of magnitude larger than the coating thickness:

$L\propto\sqrt{\alpha t}$ 2

$\rho c\frac{\partial T}{\partial t}=\nabla\cdot(k\nabla T)+Q$ 3

where the thermal diffusivity is *α* = *k*/(*cρ*); in our case, the total coating thickness is only approximately 25 μm, whereas the fused-silica substrate is 5 mm. Therefore, under the second-level irradiation conditions considered in this study, the multilayer coating becomes almost isothermal across its thickness, and most of the temperature gradient and heat storage are accommodated by the bulk substrate. Moreover, we must clarify the parameters that control the result of the heat-conduction simulations. As shown for heat conduction (**Equation 3**), the evolution of the temperature *T* is primarily governed by the density *ρ*, heat capacity *c*, conductivity *k*, and heat source *Q* (the wavelength-dependent absorption profile inside the coating was accurately obtained in this study). To quantitatively verify the impact of this simplification on the accuracy of heat conduction, we performed comparative simulations:

(1) Equivalent-layer thermal-parameter test.

We replaced the entire coating with a single layer, assigned entirely as Ta_2_O_5_ or, entirely as SiO_2_, while keeping the absorbed power distribution and all other conditions unchanged. For all three reflector designs studied, the resulting peak temperature rise at 100s changed only slightly, confirming that the overall temperature rise is weakly sensitive to the exact choice of the equivalent coating thermal parameters.

(2) Substrate-material sensitivity test.

In contrast, when we changed the fused-silica substrate to a Ta_2_O_5_ substrate (again keeping the same absorbed power distribution in the coating), the resulting peak temperature rise at 100 s decreased significantly for all designs. This clearly demonstrates that, under the conditions considered in this study, the substrate dominates the heat-transfer behavior, whereas the detailed multilayer structure plays a secondary role in the heat-conduction process.

These analyses confirm that the single-layer equivalence used in the finite-element simulations does not introduce a significant error for long-timescale heating of thin dielectric coatings on thick fused-silica substrates. We also considered interlayer thermal boundary resistance between individual coating layers. Under second-level irradiation, the thermal diffusion length far exceeded the coating thickness, meaning that any temperature discontinuity across coating interfaces rapidly disappears, rendering interfacial thermal resistance negligible for the timescales of interest. Furthermore, the coatings in this study were deposited by dual-ion beam sputtering, which is known to produce extremely dense, well-bonded oxide films with minimal interfacial defects. The AFM roughness measurements (**Figure S2**) for the three reflector designs confirm the high density and smoothness of the IBS interfaces, indicating good thermal contact between layers.

**Table S3** Peak temperature rise at 100s for the three designs with different equivalent layers and substrate.

|  | Power (W) | BRM1 (°C) | BRM2 (°C) | BRM3 (°C) |
| --- | --- | --- | --- | --- |
| Equivalent layer to that in the manuscript with fused quartz substrate | 120 | 1.400 | 1.564 | 2.250 |
|  | 150 | 2.433 | 3.723 | 6.131 |
| Equivalent Ta_2_O_5_ layer with fused quartz substrate | 120 | 1.400 | 1.564 | 2.249 |
|  | 150 | 2.433 | 3.723 | 6.130 |
| Equivalent SiO_2_ layer with fused quartz substrate | 120 | 1.406 | 1.565 | 2.256 |
|  | 150 | 2.443 | 3.747 | 6.181 |
| Equivalent layer to that in the manuscript with Ta_2_O_5_ substrate | 120 | 0.977 | 1.080 | 1.555 |
|  | 150 | 1.694 | 2.584 | 4.315 |

For the temperature simulation, a two-dimensional axisymmetric model was established. Owing to the small thickness of the multilayer film compared with that of the substrate, we simplified our computational model to a substrate with a mixed single layer consisting of half SiO_2_ and half Ta_2_O_5_. We set the boundary conditions to air convection conditions with a convective coefficient of 10 W⋅m^−2^⋅K^−1^. A laser with a Gaussian distribution was used as the pump laser with an effective beam radius of 2.35 mm. The time step was set to 0.1 ms to capture the dynamic temperature evolution caused by laser irradiation. The heat transfer module in COMSOL was used to solve Equations 9–12 in manuscript.

**Table S4** Material parameters ^[3]^

|  | *ρ* [g⋅cm^−3^] | *c* [J⋅kg^−1^⋅°C ^−1^] | *k* [W⋅m^−1^⋅°C ^−1^] |
| --- | --- | --- | --- |
| Equivalent layer | 4.5 | 525 | 2 ^[4]^ |
| SiO_2_ | 2.2 | 740 | 1.38 |
| Ta_2_O_5_ | 8.3 | 335 | 2 |
| Fused silica | 2.2 | 740 | 1.38 |

**Table S5** Fitting parameters

|  | a | b | ε_0_ |
| --- | --- | --- | --- |
| BRM1-120 W | 0.01795 ± 7.75139e−5 | 4555.97142 ± 53.54116 | 0.35586 ± 5.2529e−5 |
| BRM1-150 W | 0.01795 ± 7.75139e−5 | 4555.97142 ± 53.54116 | 0.43084 ± 6.14937e−5 |
| BRM2-120 W | 0.02185 ± 1.74509e−4 | 10917.35392 ± 205.08638 | 0.16944 ± 2.11213e−4 |
| BRM2-150 W | 0.05695 ± 5.64068e−4 | 14038.69477 ± 265.45148 | 0.19574 ± 6.3537e−4 |
| BRM3-120 W | 0.03922 ± 4.69656e−4 | 22684.03929 ± 453.03458 | 0.31554 ± 5.07736e−4 |
| BRM3-150 W | 0.08906 ± 9.6851e−4 | 22933.95248 ± 442.48673 | 0.28862 ± 0.00106 |

**Reference：**

[1] Beckmann, Petr, and Andre Spizzichino. "The scattering of electromagnetic waves from rough surfaces." Norwood (1987).

[2] Hahn, David W., and M. Necati Özisik. Heat conduction. John Wiley & Sons, 2012.

[3] R. Shah, J. Rey, and A. Stewart, “Limits of performance: CW laser damage.” SPIE. 6403. (2007). https://doi.org/10.1117/12.695918

[4] C. D. Landon, R. H. T. Wilke, M. T. Brumbach et al., “Thermal transport in tantalum oxide films for memristive applications,” Appl. Phys. Lett., 107(2), (2015). https://doi.org/10.1063/1.4926921
